# On the Use of Ergot in Certain Cases of Retention of Urine

**Published:** 1853-06

**Authors:** M. Passot

**Affiliations:** Lyons


Translated from the French for the N. W. Med. and Surg. Journal.

ART. VI.—*On the use of Ergot in certain Cases of Retention of Urine.* By M. PASSOT, of Lyons.

ERGOT possesses not only the property of exciting contractions of the uterus in cases of inertia of that organ, but is also useful in cases of retention of urine, depending on atony and paralysis of the bladder.

M. M. Baudin and Payan (of Aix) are the first who have endeavored to demonstrate that this drug does not act exclusively on the uterus, but on the inferior portion of the spinal cord. It was this that led them to its use in the affection of which we are speaking, and also in feebleness and paralysis of the inferior extremities.

Drs. Kinsley, Canuto-Canuti, Sainmont of Rocroy, and Allier of Marcigny have all presented facts, affording the strongest testimony to the utility of ergot in paralysis of the bladder. I shall present an analysis of a few cases.

Captain B., aged 60 years, had suffered from dysuria, gradually increasing for three months, when it suddenly changed to complete retention, rendering necessary the use of the catheter several times each day. During two months he was subjected to almost every variety of treatment, without the least amelioration. The prostate was enlarged, and the patient suffered severely from the introduction of the catheter, which had to be used twice each day.

50 centigrammes (gr. vijss) in a spoonful of boiling water were administered three times daily.

After about 6 hours a little urine passed and the catheter was employed but once during the day; then an interval of forty eight hours, and after six days the bladder had recovered its power.—(*Kinsley Jour. des Conn. Medico-Chir.*, *March*, 1844.)

A lady, aged about 75 years, was attacked with paralysis of the bladder, which for a long time required the use of the catheter. 16 decigrammes (gr. xxjv.) of ergot in infusion were administered. On the sixth day of the treatment the catheter was no longer necessary, the patient being able to pass her urine freely.—(*Canuto-Canuti. Bull. des Scienc. de Bologne*, 1845.)

R., aged 58 years, of a nervous temperament, was taken, after a violent attack of cholera, with complete retention of urine, so that it was necessary to relieve the bladder by the use of the catheter. The inertia of the organ continued in spite of cold injections into its interior, cold baths, the application of ice and even a blister over the hypogastric region. Strychnine ointment applied to the blistered surface, and frictions to the groins produced the next morning cramps in the legs and arms, and soon after a stiffness of the neck, which rendered it necessary to discontinue the medicine without, as yet, any action on the bladder.

At this juncture the author conceived the idea of giving ergot. He placed six *grammes*, coarsely powdered, in one *litre* of water, allowed it to stand two days, and then threw into the bladder a cold injection of this liquid. Seven minutes afterwards the patient expressed a desire to pass his urine, which, nevertheless, he was unable to do satisfactorily during the day. The next morning the injection was repeated; in eight minutes there was a desire followed by a spontaneous flow of urine. The injections were continued several days; the cure was complete.—(*Sainmont*, *Gazette des Hopitaux*, 1848.)

In 1848, Dr. Allier, of Marcigny, communicated to the National Academy of Medicine, a letter, in which he expresses the belief—the result of his observations—that in vessical paralysis the ergot will fail only one time in four. I possess also several facts to prove, in an incontestable manner, that ergot has the power to restore to the bladder its contractility.

The following is remarkable In the month of July, 1846, I was consulted by M. H., aged 60 years, a feeble constitution and a very nervous temperament.

M H. acknowledged that he had indulged in venereal excesses, to which, as a cause, he refers the paralysis of the bladder, now existing five months, and requiring twice daily the use of the catheter. There exists no organic lesion, no fever, little or no enlargement of the prostate, the urethra is free throughout its whole course, and the urine normal in quality.

After having tried, without success, frictions with tr. cantharides, and blisters to the hypogastric region, I prescribed 2 grammes of ergot recently pulverized, in 120 grammes of the *potion gommeuse* (a mixture of g. Arab., simple syrup, and water of orange flowers,) a soup spoonful to be taken every half hour, shaking the mixture each time; also the following :—

| | |
|---|---|
| ℞ Ergot pulv. | 15 decigr., |
| Butter of Cacao, | q. s., |
| M. ft. suppos, M. | ij, |

Introduce morning and evening.

The same day, but a few hours after commencing the ergot, M. H. experienced a desire to pass his urine. At my visit in the evening, I prescribed a bath; scarcely had the patient commenced bathing than micturition took place, and with force. From this time till his death the urine was free, and without the aid of a bougie. I should remark, that to complete the cure, I continued the ergot for three or four days in diminished doses.

The patient died Jan. 30th, 1848, of pleuro-pneumonia, during the course of which there was not the least symptom of a morbid condition of the bladder.

It seems, then, to be established, that ergot will cure very well retention of urine depending on atony, or pure and simple paralysis of the bladder.

In regard to paralysis following apoplexy, or dependent upon affections of the nervous centers, it is known to be difficult to cure by any medical agent whatever.

